# Paying Out-of-Pocket and Informally for Health Care in Albania: The Impoverishing Effect on Households

**DOI:** 10.3389/fpubh.2015.00207

**Published:** 2015-08-28

**Authors:** Sonila M. Tomini, Wim Groot, Milena Pavlova, Florian Tomini

**Affiliations:** ^1^Maastricht Graduate School of Governance, United Nations University-MERIT (UNU-MERIT), Maastricht University, Maastricht, Netherlands; ^2^Department of Economics, University of Liege, Liege, Belgium; ^3^Top Institute Evidence Based Education Research (TIER), Maastricht University, Maastricht, Netherlands; ^4^Department of Health Services Research, Faculty of Health, Medicine and Life Sciences, Maastricht University, Maastricht, Netherlands; ^5^Amsterdam School of Economics, University of Amsterdam, Amsterdam, Netherlands

**Keywords:** out-of-pocket expenditure, informal patient payments, poverty, Albania, catastrophic health expenditure

The health care system in Albania, as in all other ex-communist countries of Central and Eastern Europe (CEE), is rooted in the Soviet “Semashko” model. The legacies of the Semashko system still remain visible especially in the state ownership of public healthcare institutions, public provision of the services, as well as the funding from the general tax base (especially for secondary and tertiary care) (1). WHO data show that in 2013, the total health care expenditure for the country amounted to 5.9% of its GDP (2). This is relatively high compared to other former communist CEE or Former Soviet Union (FSU) countries, but still much lower than the average 8.5% for the EU15 countries (2). However, only about 48.4% of the total health care spending in Albania comes from the general state budget (2), and the share of private expenditures and out-of-pocket expenditures is relatively high (3). The utilization of health insurance in Albania remains low (4). In addition to this, almost 19% of all patients visiting outpatient services and almost 44% of patients visiting inpatient services in 2008 pay informally as well (5). But, are out-of-pocket and informal payments in Albania catastrophic to households’ budgets? If yes, what are their effects on poverty? And more importantly, what are the main policy implications for a fast-developing country like Albania?

## The Health System in Albania

The Albanian health sector during the communist period was underfinanced, and the investments in health technology were very low. The extensive web of primary health care (PHC) posts and centers and the large number of local and regional hospitals had out-dated equipment and were overstaffed (6). After the change of regime, the main reforms were focused in PHC and have sought to transfer the financing of the sector to the Health Insurance Institute (HII), which was established in 1994. The HII covers the costs of PHC visits, reimburses (part) of the drugs’ prices for drugs in the reimbursement list, as well as covers some costs of secondary and tertiary care. Ministry of Health (MoH) remains the owner and administrator of all public hospitals (4). During the past years, interventions in the hospital sector were mainly targeted to infrastructure and technology improvements and little has been done in terms of reforming the financing of providers.

Although the funding of PHC is through the HII, the sector is still dependent on subsidies from the general state budget. In 2013, about 74.1% of total public expenditure on health came from social health insurance funds while the rest came from general taxes [WHO (2)]. The health insurance contribution consists of a flat rate of 3.4% of gross salaries. However, numbers of contributors are still low due to the (still large) informal sector of the economy.

Since 2008, patients are required to pay a small fixed co-payment per visit for PHC visits or specialized treatment in hospital care (7). Despite the fact that by law all citizens should be covered by health insurance, surveys show that about 40–45% of the population declares to have a health insurance booklet (5). Previous studies have indicated that catastrophic health care payments remain high in the country (4). In fact, three main conditions are supposed to increase the incidence of catastrophic payments in health care: (i) the existence/availability of health care services requiring out-of-pocket payments, (ii) low capability from the public to pay for health care, and (iii) lack or inefficiency of the health care insurance (8). All these conditions seem to hold in Albania given that: (i) patients visiting public health centers are still required to pay out-of-pocket for many services and drugs that otherwise would be free-of-charge (5), (ii) poverty seem to be a constant concern during the last decades (9), and (iii) public health insurance is still not able to cover for all health care expenditures incurred in the public facilities (2).

## Formal and Informal Payments in Albania

Albania’s limited public spending on the health care sector (as compared to other Balkan or Eastern European countries) (10) has resulted in an increased reliance on out-of-pocket payments for both inpatient and outpatient care. Survey data report that for the lowest income quintile, the share of total out-of-pocket spending in inpatient services has gone up to 60% of the total monthly household expenditure (4). These vulnerable or poor groups of the society lack protection against out-of-pocket spending and this may contribute to increased inequalities but also to barriers to access (11). Although inpatient care is almost free for all those in possession of a health insurance booklet (except for some co-payments for high-cost diagnostic tests), in reality, most of the people visiting this service report to have paid substantial amounts of out-of-pocket payments (4). Out-of-pocket payments consist mainly of fees for services received, money to buy medicines, payments for laboratory work, transport expenditures, as well as money paid informally to medical staff. Expenses on medicines are the highest in outpatient care (12).

In general, there is a lack of clarity between formal and informal payments in Albania (4). The changes in legislation in early transition years imposed co-payments for users of PHC. Albanian health care seekers are therefore confronted with other formal out-of-pocket payments for laboratory tests, medicines, and transportation costs. However, it is not always clear whether such payments are paid formally or informally (13). As the Albanian legislation prohibits direct payments to medical staff, most of the informal payments studies focus exclusively on payments paid to medical staff. The amount paid informally to medical staff also differs (14). The main factors of this relate to attributes of patients (i.e., economic status, residence in the same locality, personal relations, and societal/political position) attributes of providers (specialists vs. general practitioners, highly specialized medical staff, and availability), the type of services (inpatient/outpatient, locality, specialty, complexity of treatment, and technology involved), and other contextual factors (like urbanization of the locality, social norms, etc) (5, 14). Payment mechanisms also tend to differ and are complex. Despite the illegal nature of such payments, they are reported to take place in the open and are often not something that is hidden. Patients may gather information from social networks but in many cases the nurses or physicians directly induce the payments. Some of these strategies involve talking about the low salaries, leaving money on the table (to show that others have also paid), requesting them from patients or relatives accompanying the patient, acting unfriendly, or delaying care (14). The impact of these payments on patient’s welfare has proven to be quite substantial and the situation is particularly dramatic for people in the lowest quintile of the expenditure distribution (15).

## Are Out-of-Pocket Payments in Albania Catastrophic for Households’ Budgets?

Out-of-pocket expenditures for health care can be a heavy burden on household’s expenditures. If they are too high, they can also hinder household’s long-term income generating capabilities. Out-of-pocket expenditures for health care are considered catastrophic when they force individuals or households to significantly decrease their standard of living now or in the future (16). This pushes them not only into a closed circle of inter-generational transmission of poverty (17) but may also prevent them from getting necessary health care treatment.

A recent study (4) shows that payments per health care episode constitute a substantive share of total monthly per capita expenditures. When looking at the share of out-of-pocket expenditures over total non-health expenditures and using a 10% threshold to define a catastrophic health care payment for that household, almost 22.6% of the population had catastrophic out-of-pocket payments in 2002, while this incidence declined in 2005 and 2008 to, 17.6 and 13.3%, respectively. Despite this decrease, the incidence of catastrophic out-of-pocket payments remains high, and moreover, this is higher for vulnerable groups of the population. Evidence from the same study (4) shows that for the lowest quintile, this incidence declined by a lower extent for the poorest quintile, i.e., from 29.9% in 2002 to 28.7% in 2005 and 20% in 2008.

**Figure 1:**
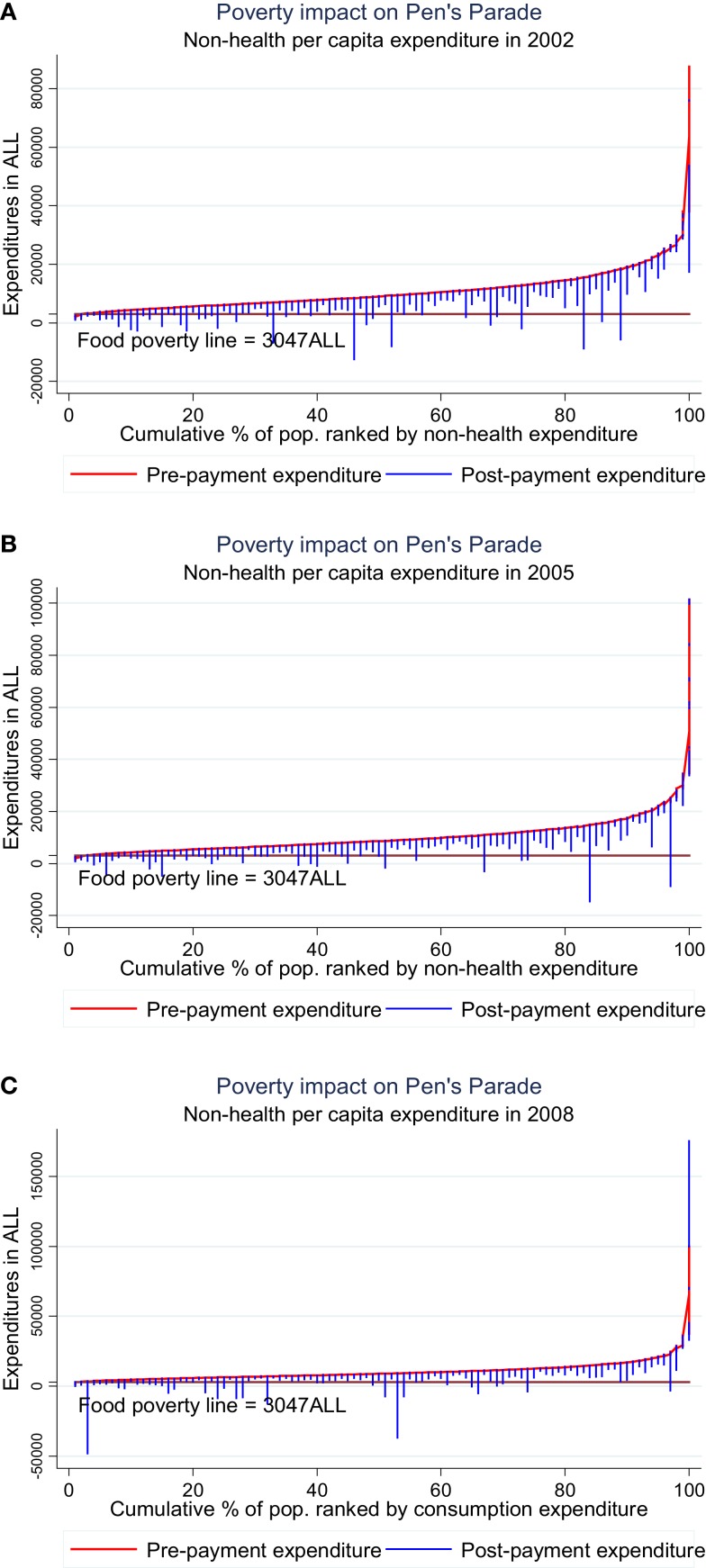
**Poverty impact of health expenditure on the distribution of non-health expenditure**. **(A)** Year 2002, **(B)** year 2005, and **(C)** year 2008. Source: Tomini et al. (4).

In fact, the effect of catastrophic out-of-pocket payments is most worrying if it pushes households in poverty. The pre-payment and post-payment poverty headcount rates can tell about this effect. Jan Pen’s parade of “dwarfs and a few giants” (18) depicts total household expenditures with and without (gross and net) of total out-of-pocket payments and helps to visualize this (see Figure 1).

The graphs show clearly that the effect of out-of-pocket payments may be catastrophic (i.e., push households below the food poverty line of 2 US$ a day) and that this is not only observed for the poorest quintiles. The graphs show also clearly that an increase in formal or informal payments can be problematic even for the highest quintiles in the absence of insurance to compensate for the financial losses.

## Limitations to Studying Catastrophic Impact of Out-of-Pocket Payments

One of the main limitations in studying the impoverishing effect of out-of-pocket and informal payments is the lack of information on those patients that needed health care but could not afford it. Survey data give information only on patients that have sought health care and do not allow estimating the gap that needs to be filled in order to ensure equal access for everyone. Other limitations relate to the most likely underestimated effect of informal payments. Survey data for Albania allow distinguishing only the part of informal payments paid as “gifts” to medical staff. Other definitions of informal payments may include more types of informal payments. Additional data (allowing for a more comprehensive definition of informal payments) may provide more insights on the overall causes of informal payments and the burden imposed on households. Also household surveys are not necessarily randomized based on health and health care-related information. This may lead to an underrepresentation of certain groups (especially high utilization groups like the elderly or chronically ill) and therefore underestimate the effect of out-of-pocket payments for such groups.

## Policy Implications

The existence of catastrophic health care expenditures raises concern. Catastrophic health care expenditures do not only impose a higher poverty risk for people seeking health care but may also impose barriers to access for them (19). The Albanian authorities should seriously consider the reduction of total out-of-pocket payments, which amount to almost 60% of total expenditures for health care in the country. This is best achieved through ensuring the effectiveness and attractiveness of formal mechanisms of health care financing (i.e., general tax revenues and health care insurance). While improving the effectiveness of such mechanisms requires a better coordination and allocation of resources, the attractiveness could be raised by adopting the structure of contributions and co-payments so that they better reflect the income distribution. Measures like fee exemptions or price subsidies for vulnerable groups have already proven effective in reducing catastrophic payments in other countries (20).

Other measures like subsidized transportation for the poor or a better distribution of health care centers would also help in this regard. But, on the other side, any policy reform aiming to increase health care utilization of the poor should evaluate the effect on catastrophic payments, especially for the poor and the vulnerable. Previous research has warned that focusing only on the availability of health services can indeed contribute to improving health of the poor but it may also increase the proportion of poor households facing catastrophic expenditures (8).

Further research should be focused on identifying the effect of out-of-pocket and informal payments on people who cannot afford such payments and are therefore denied access to health care. In fact, previous research has shown that more that from the effect of catastrophic health care expenditures, the poor suffers the catastrophic effect of illness given the barriers to access and the consequences on the uninsured shocks on prospective incomes from employment (19). Another interesting aspect for future research is also the investigation of the effectiveness of policy measures, like fee exemptions and price subsidies, in reducing the risk of falling in poverty among particular health care seekers addressed by these policies.

## Conflict of Interest Statement

The authors declare that the research was conducted in the absence of any commercial or financial relationships that could be construed as a potential conflict of interest.
